# Platelet Inhibition by Nitrite Is Dependent on Erythrocytes and Deoxygenation

**DOI:** 10.1371/journal.pone.0030380

**Published:** 2012-01-20

**Authors:** Sirada Srihirun, Thanaporn Sriwantana, Supeenun Unchern, Dusadee Kittikool, Egarit Noulsri, Kovit Pattanapanyasat, Suthat Fucharoen, Barbora Piknova, Alan N. Schechter, Nathawut Sibmooh

**Affiliations:** 1 Department of Pharmacology, Faculty of Science, Mahidol University, Bangkok, Thailand; 2 Office for Research and Development, Faculty of Medicine Siriraj Hospital, Mahidol University, Bangkok, Thailand; 3 Thalassemia Research Center, Institute of Science and Technology for Research and Development, Mahidol University, Nakhonpathom, Thailand; 4 Molecular Medicine Branch, National Institute of Diabetes and Digestive and Kidney Diseases, National Institutes of Health, Bethesda, Maryland, United States of America; University of Frankfurt - University Hospital Frankfurt, Germany

## Abstract

**Background:**

Nitrite is a nitric oxide (NO) metabolite in tissues and blood, which can be converted to NO under hypoxia to facilitate tissue perfusion. Although nitrite is known to cause vasodilation following its reduction to NO, the effect of nitrite on platelet activity remains unclear. In this study, the effect of nitrite and nitrite+erythrocytes, with and without deoxygenation, on platelet activity was investigated.

**Methodology/Finding:**

Platelet aggregation was studied in platelet-rich plasma (PRP) and PRP+erythrocytes by turbidimetric and impedance aggregometry, respectively. In PRP, DEANONOate inhibited platelet aggregation induced by ADP while nitrite had no effect on platelets. In PRP+erythrocytes, the inhibitory effect of DEANONOate on platelets decreased whereas nitrite at physiologic concentration (0.1 µM) inhibited platelet aggregation and ATP release. The effect of nitrite+erythrocytes on platelets was abrogated by C-PTIO (a membrane-impermeable NO scavenger), suggesting an NO-mediated action. Furthermore, deoxygenation enhanced the effect of nitrite as observed from a decrease of P-selectin expression and increase of the cGMP levels in platelets. The ADP-induced platelet aggregation in whole blood showed inverse correlations with the nitrite levels in whole blood and erythrocytes.

**Conclusion:**

Nitrite alone at physiological levels has no effect on platelets in plasma. Nitrite in the presence of erythrocytes inhibits platelets through its reduction to NO, which is promoted by deoxygenation. Nitrite may have role in modulating platelet activity in the circulation, especially during hypoxia.

## Introduction

Nitrite is recognized as a bioactive NO metabolite and is involved in the regulation of vascular tone by induction of vasodilation during ischemia. The effect of nitrite appears to be mediated through NO which activates soluble guanylyl cyclase to produce cGMP, leading to vasodilation [1], [2]. The nitrite reductase activity of deoxygenated heme-containing proteins especially hemoglobin, and other pathways including the reactions of xanthine oxidoreductase under hypoxia and acidosis are believed to be responsible for the reduction of nitrite to NO, which contributes to the mechanism of hypoxic vasodilation [3], [4], [5].

In addition to vasodilation, NO inhibits platelet reactivity [6]. The effect of NO on platelets is mediated through the activation of guanylyl cyclase to produce cGMP [7]. Although the vasodilatory effect of nitrite has been widely studied, the effect of nitrite on platelet activity has received little attention. Nitrite at high concentration (500 µM) was reported to inhibit ADP-induced platelet aggregation and increase the cGMP levels in plasma [8]. This effect of nitrite on platelets is likely to be through NO and the subsequent activation of guanylyl cyclase. The elevation of plasma nitrite levels after the ingestion of nitrate-rich diet reduces the blood pressure and attenuates the platelet aggregation induced by ADP and collagen in plasma, suggesting a possible physiological role of nitrite on platelet activity [9]. Nevertheless, any contribution of the expected effect of erythrocytes and deoxygenation on the action of physiological levels of nitrite on platelets remains to be elucidated.

We hypothesized that the effects of nitrite on platelets would require erythrocytes and that these effects could be enhanced by the deoxygenation of erythrocytes. By using the impedance aggregometry and flow cytometric measurement of P-selectin expression, we demonstrate the inhibitory effect of nitrite+erythrocytes on platelets. An inverse correlation of nitrite levels in blood and the ADP-induced platelet aggregation in whole blood is also shown. However, erythrocytes also have ability to destroy NO and this leads to apparent contradictory effects of nitrite and NO donors on platelet aggregation.

## Results

### Nitrite had no effect on platelet aggregation in PRP

**Figure 1:**
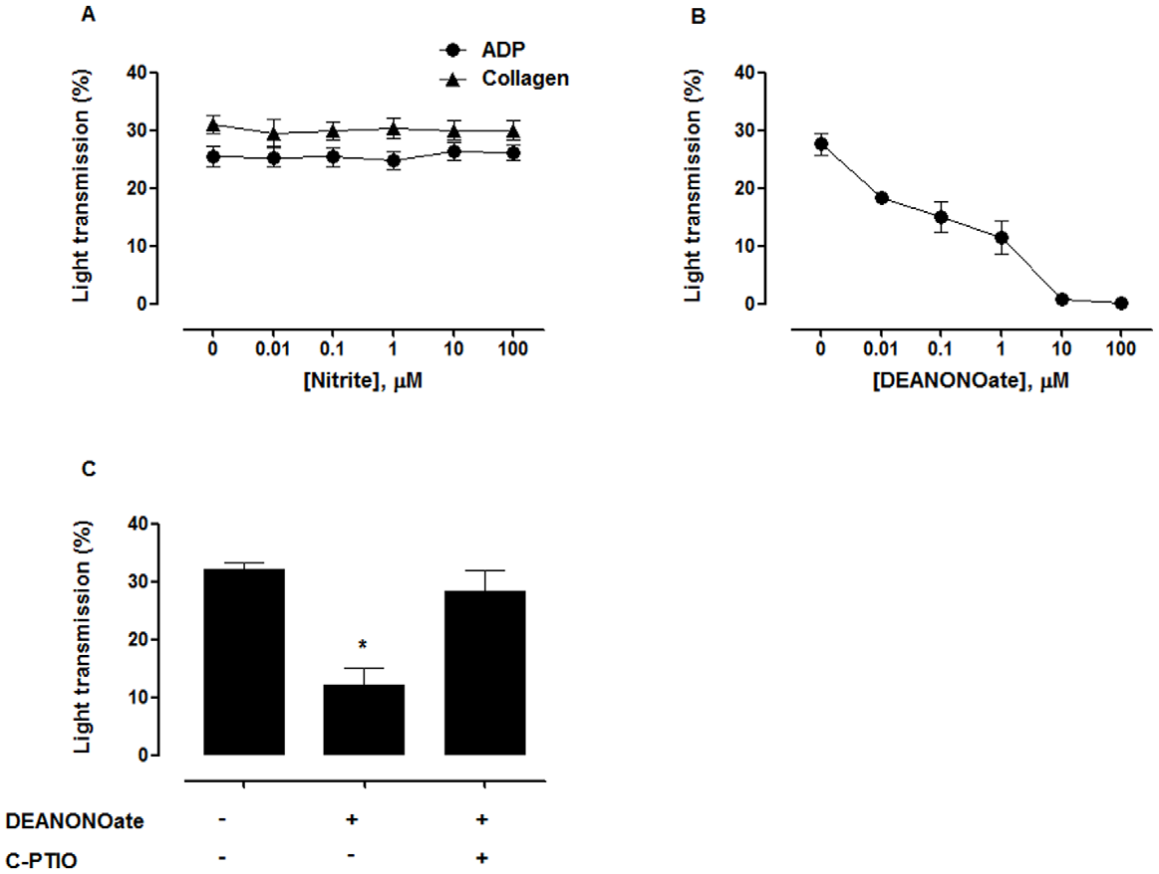
Nitrite had no effect on platelet aggregation in plasma. (A) Nitrite had no effect on ADP and collagen-induced platelet aggregation. Nitrite was pre-incubated in PRP for 5 min. Platelet aggregation was induced by 8 µM ADP or 2.5 µg/mL collagen. (B) DEANONOate inhibited ADP-induced platelet aggregation. DEANONOate was pre-incubated in PRP for 5 min. Platelet aggregation was induced by 8 µM ADP. (C) C-PTIO inhibited the anti-platelet activity of DEANONOate. PRP was incubated with 1 µM DEANONOate and 200 µM C-PTIO for 5 min before the induction of aggregation by 8 µM ADP. * *P*<0.05 compared with the others (ANOVA). All experiments were performed at 37°C. Data are means ± SEM (n≥3).

Nitrite even at the supraphysiologic concentrations up to 100 µM incubated in PRP for 5 min did not inhibit platelet aggregation induced by ADP or collagen (Figure 1A). In contrast, DEANONOate inhibited ADP-induced platelet aggregation (Figure 1B). 200 µM C-PTIO (an NO scavenger) [10] blocked the effect of 1 µM DEANONOate (Figure 1C). Co-incubation of ascorbic acid, a reducing agent even at concentrations up to 1 mM, with nitrite did not inhibit aggregation in PRP (data not shown).

### Effects of erythrocytes on inhibition by nitrite and DEANONOate of platelet aggregation and ATP release

**Figure 2:**
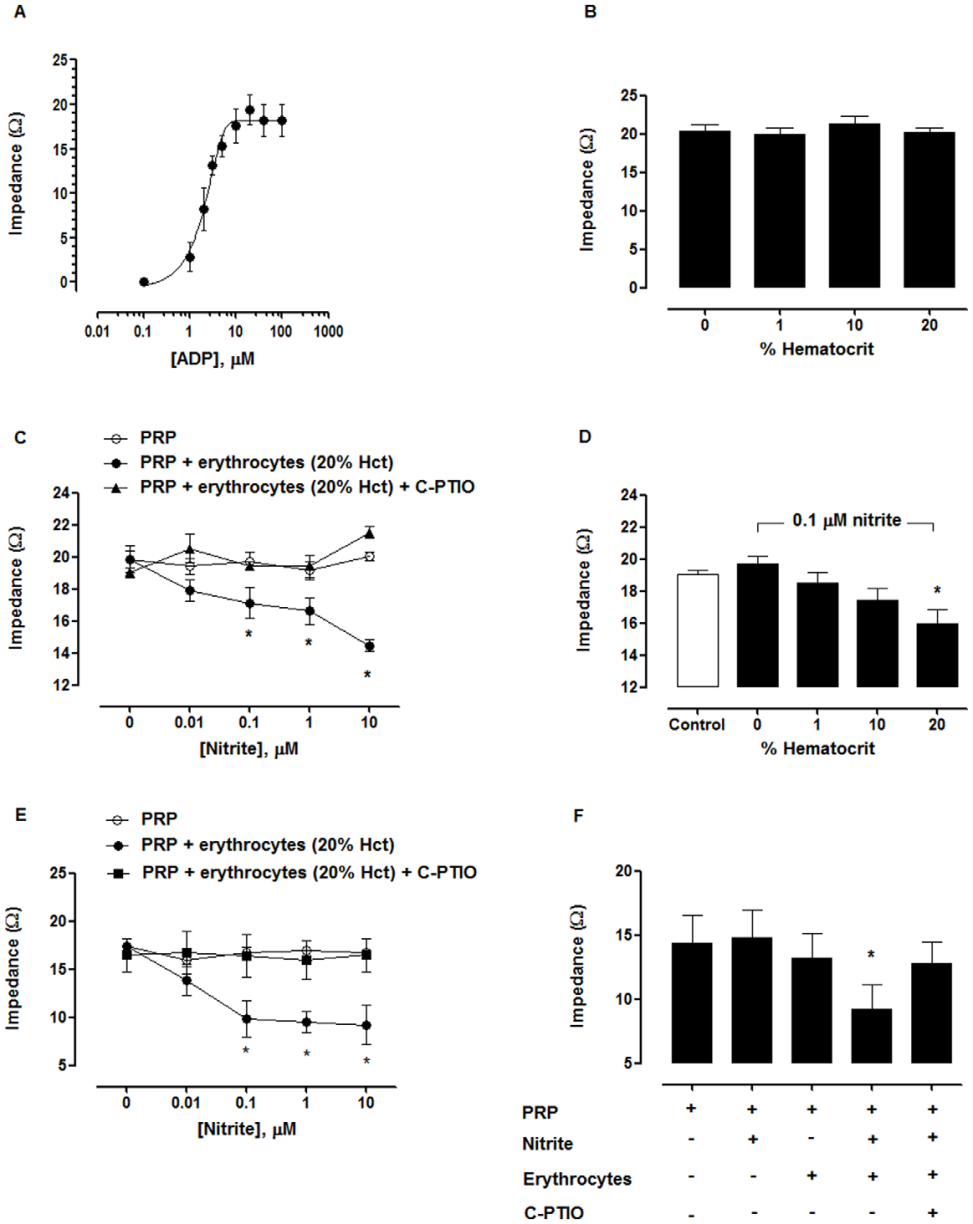
Nitrite+erythrocytes inhibited platelet aggregation. (A) ADP induced platelet aggregation in PRP+erythrocytes (20% hematocrit) in the concentration dependent manner. (B) Erythrocytes at 0, 1, 10 and 20% hematocrit did not have effect on ADP-induced platelet aggregation in the absence of nitrite. (C) Nitrite+erythrocytes (20% hematocrit) inhibited ADP-induced platelet aggregation. Nitrite was incubated in PRP or PRP+erythrocytes in the presence or absence of 200 µM C-PTIO for 5 min before induction of aggregation by ADP. ^*^ *P*<0.05 compared with PRP and PRP+erythrocytes+C-PTIO (ANOVA). (D) The inhibitory effect of 0.1 µM nitrite was dependent on hematocrits. PRP and PRP+erythrocytes (1, 10, and 20% hematocrit) were incubated with 0.1 µM nitrite for 5 min before induction of platelet aggregation by 20 µM ADP. ADP-induced platelet aggregation in the absence of nitrite was shown in the control group. ^*^ *P*<0.05 compared with PRP+nitrite at 0% hematocrit (ANOVA). (E) Nitrite+erythrocytes inhibited collagen-induced platelet aggregation. PRP or PRP+erythrocytes samples (20% hematocrit) were incubated with nitrite in the presence and absence of 200 µM C-PTIO for 5 min and then the aggregation was induced by 2.5 µg/mL collagen. ^*^ *P*<0.05 compared with PRP and PRP+erythrocytes+C-PTIO (ANOVA). (F) Nitrite+erythrocytes inhibited U46619-induced platelet aggregation. 0.1 µM nitrite was incubated in PRP or PRP+erythrocytes (20% hematocrit) in the presence or absence of 200 µM C-PTIO for 5 min. Then, the aggregation was induced by 1 µM U46619. ^*^ *P*<0.05 compared with PRP+erythrocytes and PRP+erythrocytes+C-PTIO (ANOVA). All experiments were performed at 37°C. Data are means ± SEM (n≥3).

Because erythrocytes could contribute to nitrite reduction to NO [11], we tested whether an inhibition of aggregation would be observed after addition of erythrocytes to PRP. The impedance aggregometry method was used for the mixtures of PRP+erythrocytes because the erythrocytes interfered with the turbidimetric method. In this condition, 10–20 µM ADP produced the maximal aggregation (Figure 2A); thus, we used 20 µM ADP in the subsequent experiments of PRP+erythrocytes. In the absence of nitrite, platelet aggregation was not different among PRP+erythrocytes at 0, 1, 10 and 20% hematocrits (Figure 2B).

The incubation of nitrite (0.01–10 µM) in PRP+erythrocytes (20% hematocrit) decreased ADP-induced platelet aggregation. In PRP+erythrocytes at 20% hematocrit, nitrite at 0.1–10 µM inhibited aggregation significantly (EC_50_ = 1.3±0.6 µM, maximum inhibition = 26.7±2.7%) (Figure 2C). 200 µM C-PTIO inhibited the effect of nitrite+erythrocytes on platelet aggregation. C-PTIO alone had no effect on platelet aggregation induced by ADP. In PRP+erythrocytes at 20% hematocrit, nitrite at physiologic concentration (0.1 µM) decreased platelet aggregation from 19.0±0.3 to 15.9±0.9 Ω (*P*<0.05 compared with the value at 0% hematocrit) (Figure 2D). Thus, the effect of 0.1 µM nitrite was dependent on the hematocrit.

The effect of nitrite on platelet aggregation induced by collagen and U46619 (thromboxane A_2_ agonist) was also examined. Similar to the ADP experiments, nitrite alone did not inhibit platelet aggregation induced by collagen or U46619 in plasma; however, in the presence of erythrocytes (20% hematocrit), 0.1 µM nitrite inhibited platelet aggregation. In addition, the effect of nitrite was diminished in the presence of C-PTIO (Figure 2E, F).

**Figure 3:**
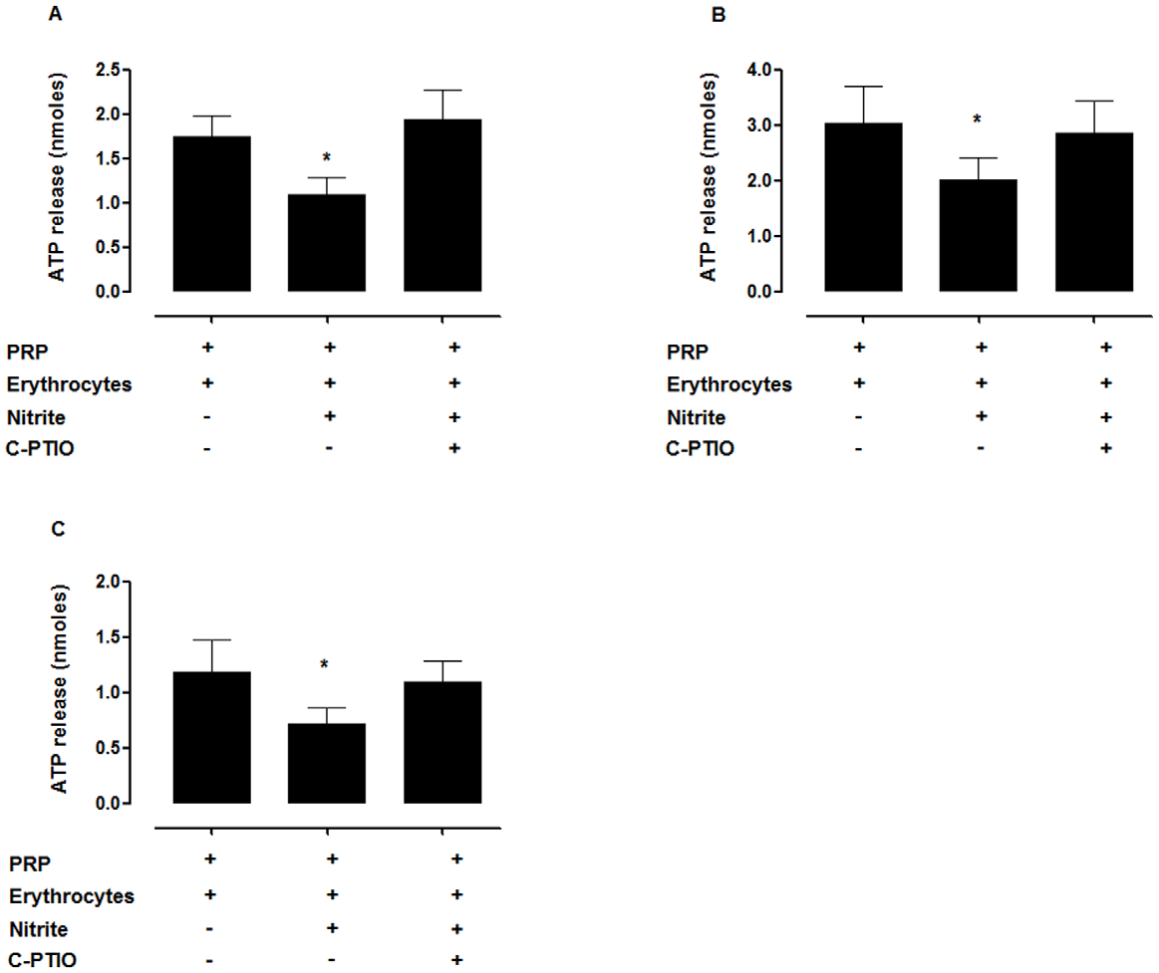
Nitrite+erythrocytes inhibited ATP release from platelets induced by ADP (A), collagen (B), and U46619 (C). PRP+erythrocytes (20% hematocrit) were incubated with 0.1 µM nitrite in the presence and absence of 200 µM C-PTIO for 5 min. The ATP release was induced by 20 µM ADP, 2.5 µg/mL collagen or 1 µM U46619. ^*^ *P*<0.05 compared with PRP+erythrocytes and PRP+erythrocytes+nitrite+C-PTIO (ANOVA). All experiments were performed at 37°C. Data are means ± SEM (n = 5).

Apart from aggregation, nitrite+erythrocytes also inhibited ATP release from platelets. In the presence of erythrocytes, 0.1 µM nitrite decreased the ATP release induced by 20 µM ADP, 2.5 µg/mL collagen, and 1 µM U46619. C-PTIO completely inhibited the effect of nitrite+erythrocytes (Figure 3A–C).

**Figure 4:**
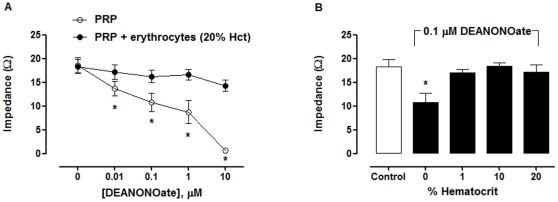
Erythrocytes abolished the effect of DEANONOate on platelet aggregation. (A) Inhibition of platelet aggregation by DEANONOate decreased in the presence of erythrocytes. PRP and PRP+erythrocytes (20% hematocrit) were incubated with DEANONOate for 5 min. (B) Dependence of platelet aggregation in the presence of 0.1 µM DEANONOate on hematocrit. PRP and PRP+erythrocytes (1, 10, and 20% hematocrit) were incubated with 0.1 µM DEANONOate for 5 min. Platelet aggregation was induced by 20 µM ADP. ADP-induced platelet aggregation in the absence of nitrite was shown in the control group ^*^ *P*<0.05 compared with 0% hematocrit (ANOVA). All experiments were performed at 37°C. Data are means ± SEM (n≥3).

In contrast to nitrite, the inhibitory effect of DEANONOate on platelet aggregation decreased in the presence of erythrocytes. Erythrocytes at 1% hematocrit attenuated the effect of DEANONOate (Figure 4A, B), presumably due to NO destruction by hemoglobin in erythrocytes.

### Deoxygenation of erythrocytes enhanced the inhibitory effect of nitrite on platelet activation

**Figure 5:**
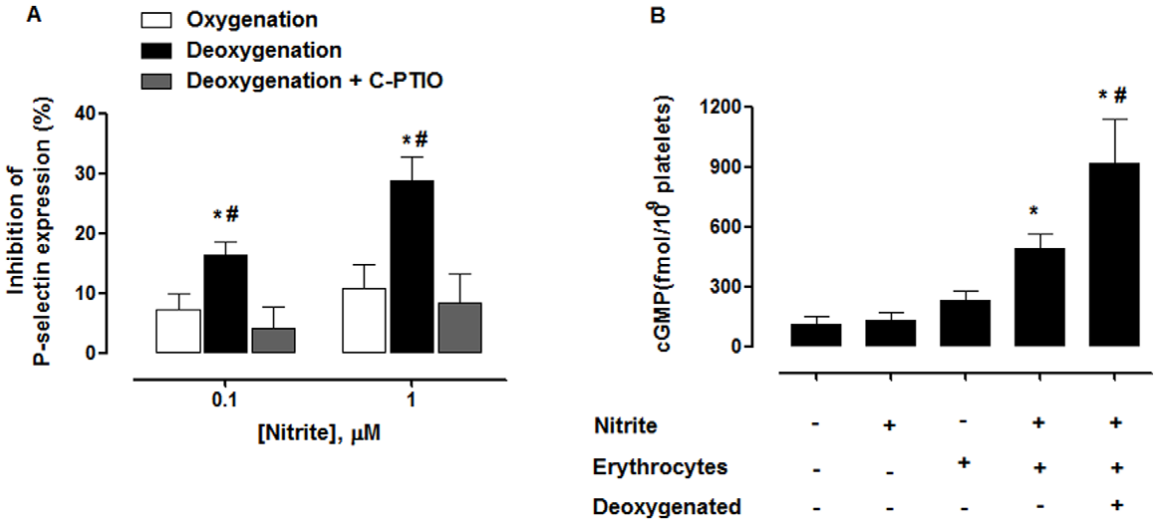
Deoxygenation enhanced platelet inhibition by nitrite+erythrocytes. PRP was pre-incubated with nitrite (0.1 or 1 µM) or nitrite+C-PTIO (200 µM) in the presence of oxygenated or deoxygenated erythrocytes (20% hematocrit) for 5 min. Then, 20 µM ADP was added to the cell suspension and incubated for 10 min. Platelet activation was determined by the flow cytometry as an expression of P-selectin. ^*^ *P*<0.05 compared with the values of oxygenated erythrocytes. ^#^ *P*<0.05 compared with deoxygenated erythrocytes+C-PTIO (ANOVA). (B) Nitrite+erythrocytes increase the cGMP levels in platelets. Washed platelets+erythrocyte samples (20% hematocrit) were incubated with 0.1 µM nitrite for 5 min. ^*^ *P*<0.05 compared with platelets. ^#^ *P*<0.05 compared with platelets+nitrite+erythrocytes (ANOVA). All experiments were performed at 37°C. Data are means ± SEM (n≥4).

Further, we investigated whether the deoxygenation could enhance the effect of nitrite+erythrocytes on platelet activity. In the presence of cell-free hemoglobin, the effect of nitrite on platelets was abolished because nitrite reacted rapidly with free hemoglobin to form the inactive nitrate ions [12]. Thus, the washed erythrocytes were prepared freshly to avoid any cell-free hemoglobin. The erythrocyte suspension was deoxygenated by helium gas and added into the platelet suspension. Activation of platelets was induced by 20 µM ADP and determined by the flow cytometry as an expression of P-selectin (CD62P). The percentage of platelets that expressed P-selectin before addition of ADP was 0.12±0.06. After addition of 20 µM ADP, the percentage of platelets that expressed P-selectin increased to 16.2±3.7 and 16.6±4.2 in the presence of oxygenated and deoxygenated erythrocytes, respectively. Thus, the degree of platelet activation under oxygenated and deoxygenated condition was not different. In the presence of erythrocytes (at room air, PO_2_∼57 mmHg), 0.1 and 1 µM nitrite inhibited P-selectin expression. In the presence of deoxygenated erythrocytes (PO_2_∼25 mmHg), the effect of 0.1 and 1 µM nitrite increased further to 16.3±2.4 and 28.7±4.1% inhibition of P-selectin expression, respectively (Figure 5A). C-PTIO blocked the effect of nitrite+erythrocytes on P-selectin expression in the deoxygenated condition. Similarly, nitrite alone (0.1 µM) had no effect on the cGMP levels in platelets. Nitrite+erythrocytes increased the cGMP levels in platelets, and deoxygenation caused further increase in the cGMP levels (Figure 5B). These results also suggest that the platelet inhibition by nitrite+erythrocytes was enhanced further by deoxygenation.

### Platelet aggregation in whole blood of volunteers was correlated with the nitrite levels

**Figure 6:**
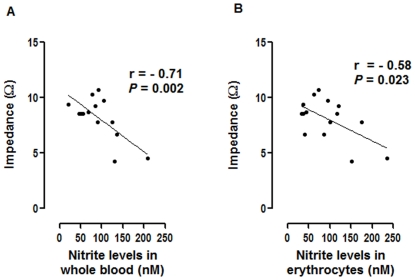
Platelet aggregation in whole blood showed inverse correlation with the nitrite levels in whole blood and erythrocytes. Platelet aggregation in whole blood (20% hematocrit) was induced by 10 µM ADP and monitored for 5 min using the impedance aggregometry. The degree of platelet aggregation was correlated with the nitrite levels in whole blood (A) and erythrocytes (B). Each dot represents mean of duplicate measurement (n = 15).

To study the potential role of nitrite in the regulation of platelet activity, we investigated the correlation between the nitrite levels in blood from 15 healthy donors and platelet aggregation in whole blood using the impedance aggregometry. The levels of nitrite in blood were measured by tri-iodide based chemiluminescense. Platelet aggregation in whole blood (20% hematocrit) was induced by 10 µM ADP and monitored by impedance aggregometry. The means ± SEM of the nitrite levels in whole blood, erythrocytes and plasma were 85.6±10.9, 93.4±15.1 and 47.2±7.0 nM, respectively. The nitrite levels in platelets were 13.6±4.8 nmol/10^12^ cells. We found the inverse correlations between the extent of ADP-induced platelet aggregation in whole blood at 5 min and the nitrite levels in whole blood and erythrocytes (Figure 6A, B). However, there was no significant correlation between platelet aggregation and the nitrite levels in either plasma or the platelets.

## Discussion

Our results demonstrate the inhibitory effect of nitrite+erythrocytes on platelets, but nitrite alone at physiological concentrations has no effect on platelets. The effect of nitrite+erythrocytes on platelet aggregation is inhibited by an NO scavenger, suggesting that the effect is mediated through NO. Furthermore, deoxygenation promotes the effect of nitrite+erythrocytes and causes further increase of the cGMP levels in platelets. This is likely caused by the nitrite reductase activity of deoxygenated hemoglobin while a reducing agent such as ascorbic acid did not have this effect. The inverse correlations between the nitrite levels in whole blood and erythrocytes with platelet aggregation in volunteers raise the possibility that nitrite may be involved in modulating the baseline platelet activity.

From our results, we conclude that nitrite itself does not inhibit platelet aggregation induced by ADP, collagen and U46619 in plasma. This is consistent with a previous study [8] which reports that nitrite only at high concentration (500 µM) inhibits platelet aggregation induced by ADP in plasma. However, in the presence of erythrocytes at 20% hematocrit, the inhibitory activity of nitrite becomes demonstrable whereas that of DEANONOate decreases. Because the erythrocytes at hematocrit greater than 20% would interfere with the impedance aggregometry, the PRP+erythrocytes at this hematocrit value was used in the subsequent studies. The 20% hematocrit, however, is thought to occur in most vessels of the microcirculation where much thrombosis occurs and could be found in large vessels under anemic conditions such as thalassemia or malaria.

The inhibitory effect of DEANONOate on platelets decreases in the presence of erythrocytes. This suggests that NO released from DEANONOate may diffuse rapidly into erythrocytes and then react with hemoglobin to form inactive nitrate and methemoglobin (*k* = 5×10^7^ and 2.6×10^7^ M^−1^.s^−1^ for the reaction of NO with oxy- and deoxyhemoglobin, respectively) [13], [14]. This suggests that the scavenging of NO by hemoglobin in the erythrocytes may be responsible for the decreased effect; which is in agreement with the reports that NO donors such as DEANONOate and S-nitrosothiol have decreased ability to inhibit platelets in whole blood [15]. Thus, NO dissociated from NO donor would be taken up into erythrocytes rapidly and would be unable to escape from erythrocytes; in contrast to the mechanisms apparently operative when the NO is generated in the erythrocytes themselves (see below).

The reaction of nitrite with deoxyhemoglobin would generate NO because of the nitrite reductase activity of deoxyhemoglobin [16], [17]. Nitrite may enter the erythrocytes in the form of HNO_2_ via simple or facilitated diffusion through the anion exchanger-1 (AE-1) [18], [19], [20]. Because deoxyhemoglobin has higher affinity than oxyhemoglobin in binding AE-1 [21], nitrite is proposed to react with deoxyhemoglobin located near the cytoplasmic surface of the erythrocytic membrane. Moreover, the reaction of nitrite with deoxyhemoglobin bound to AE-1 is faster than that with free deoxyhemoglobin [22]. Nitrite in erythrocytes reacts with deoxyhemoglobin to form the iron-nitrosyl-hemoglobin (HbNO) which is unstable [23], [24]. The formation of HbNO is enhanced under conditions of reduced oxygen saturation. The HbNO could be oxidized easily by intracellular oxidants such as dehydroascorbic acid [24], producing NO or related compounds. Although most nitrite and NO is scavenged by hemoglobin inside erythrocytes, some nitrite may react with the deoxyhemoglobin molecules located near the membrane, leading to the release of NO or an intermediate such as N_2_O_3_ from erythrocytes [25]. However, other mechanisms are possible because of the complex kinetics of many processes involved.

To examine whether the effect of nitrite was through NO, C-PTIO was used to inhibit the effect of nitrite on platelet aggregation. Our results show that C-PTIO diminished the inhibitory effect of nitrite on platelet aggregation, ATP release and P-selectin expression. These are consistent with the previous evidence that vasodilation induced by nitrite in the presence of erythrocytes is mediated through the generation of extracellular NO [11]; which is in accordance with the mechanism proposed above.

The reports that vasodilation induced by nitrite is dependent on erythrocytes and that deoxyhemoglobin promotes the vasodilatory effect of nitrite by conversion of nitrite to NO [3], [11], led us to hypothesize further that the deoxygenated erythrocytes would be involved in the mechanism of platelet inhibition by nitrite. The inhibitory effect of nitrite+erythrocytes on platelets could be increased under deoxygenated condition because deoxyhemoglobin possesses the nitrite reductase activity. Our results demonstrate that deoxygenation enhances the inhibitory effect of nitrite+erythrocytes on platelets as observed from a decrease of P-selectin expression but increase of cGMP levels. Because nitrite does not activate guanylyl cyclase [26], an increase in cGMP levels in platelets could result from NO. Therefore, these suggest that NO produced from the interaction of nitrite with deoxyhemoglobin may contribute to platelet inhibition. Apart from deoxyhemoglobin, other heme-containing proteins and enzymes, such as xanthine oxidoreductase, eNOS, and cytochrome P-450 oxidoreductase, could be involved in the reduction of nitrite to NO [27], [28].

The correlations between platelet aggregation and nitrite levels in whole blood and erythrocytes of volunteers suggest the role of nitrite as one of several factors regulating platelet activity in vivo. In our experiments, the nitrite levels were measured in fasting blood to avoid the influence of diet. Despite using the nitrite-preserving solution, the nitrite concentrations in whole blood, erythrocytes, and plasma were somewhat lower than those reported previously for different populations and under different conditions [29]. Further studies on variation in the nitrite levels in different population and age groups, and the effects of diet and other factors remain to be investigated.

In addition, the correlation of blood nitrite and platelet aggregation also raises the possibility of direct in vivo interactions between the erythrocytes and platelets. Platelet activity would be regulated by NO that is produced outside platelets and not from eNOS within the platelets [30]. The erythrocytes are the major storage site of nitrite in blood. The erythrocytic nitrite has a half-life of 10 min in whole blood after drawing [29] and the nitrite levels would be related to the endothelial eNOS activity via endothelial function [31]. Thus, the erythrocytic nitrite could regulate platelet activity by providing NO, especially under hypoxia. The decreased nitrite levels in blood, as expected in hemolytic diseases such as sickle cell disease or thalassemia where the cell-free hemoglobin would react with nitrite or NO, would be expected to be associated with platelet hyperactivity and vascular complications [32].

In conclusion, nitrite does not have a direct inhibitory effect on platelets when studied in plasma. Nonetheless, nitrite+erythrocytes inhibit platelets through the nitrite reduction to NO; which is promoted in the deoxygenated condition. Together with vasodilation, the inhibition of platelets by nitrite+erythrocytes could promote blood flow under hypoxic conditions.

## Materials and Methods

### Ethics statement

This study was approved by the Ramathibodi Hospital Ethics Committee. The written informed consent was obtained in accordance with the Declaration of Helsinki.

### Materials

Sodium nitrite (NaNO_2_), adenosine 5′diphosphate (ADP), collagen, U46619 were purchased from Sigma (St Louis, MO). Diethylamine diazeniumdiolate (DEANONOate) was purchased from Cayman Chemical (Ann Arbor, MI). 2-(4-carboxyphenyl)-4,4,5,5-tetramethylimidazole-1-oxyl 3-oxide (C-PTIO) was purchased from Alexis (Lausen, Switzerland). Luciferin-luciferase reagent (Chrono-Lume) was purchased from Chrono-Log Corporation (Howertown, PA). DEANONOate was freshly prepared by dissolving in 0.01 M NaOH and used within 1 day. Immediately before use, DEANONOate was diluted in phosphate-buffered saline (PBS, pH 7.4). Sodium nitrite, ADP, and C-PTIO were prepared in PBS at pH 7.4. Collagen was prepared in deionized water.

Monoclonal antibodies: FITC-labeled anti-human CD41a and PE-labeled anti-human CD62P were purchased from Becton Dickinson (San Jose, CA).

### Healthy volunteers

The volunteers had been in fasting state for at least 8 h. The fasting venous blood samples were taken from healthy volunteers, of 28.0±4.1 (mean ± SD) years of age, and placed in tubes containing 3.8% sodium citrate in the ratio of 9∶1 (vol/vol). The volunteers were non-smokers and had not taken nonsteroidal anti-inflammatory drug within a week or other medications within 48 h.

### Platelet aggregation and ATP release

Whole blood was centrifuged at 120×g for 10 min at room temperature. The upper portion was collected for PRP. The lower portion was centrifuged further at 2000×g for 10 min to obtain the platelet-poor plasma (PPP). The PRP was used for aggregation studies within 2 h after blood drawing. The effect of sodium nitrite on platelet aggregation in PRP and PRP+erythrocytes was determined by the turbidimetric and impedance aggregometry, respectively.

In turbidimetric experiments, the PRP was pre-incubated with 0.01–100 µM sodium nitrite or DEANONOate at 37°C for 5 min. In separate experiments, the PRP was pre-incubated with 1 µM DEANONOate for 5 min in the presence or absence of 200 µM C-PTIO. Platelet aggregation was induced by 8 µM ADP or 2.5 µg/mL collagen. ADP at 8 µM, which was the lowest concentrations that caused irreversible platelet aggregation, was used in the subsequent experiment in PRP. Platelet aggregation was monitored by the Aggregometer Model 500/560 CA (Chrono-Log Corporation, Howertown, PA) for 4 min at 37°C. The increased light transmission indicated increased aggregation in PRP.

In impedance experiments, platelet aggregation in PRP or PRP+erythrocytes was studied by impedance aggregometry in which the change of electrical impedance (in ohm, Ω) was monitored. The increase of the electrical impedance of electrode correlated with the aggregation. The aggregation was induced by 20 µM ADP, 2.5 µg/mL collagen or 1 µM U46619 and recorded for 5 min at 37°C. Because the pre-incubation of nitrite+erythrocytes in PRP for 5 min caused the stable maximal inhibition of aggregation, the 5-min duration of incubation was used in the subsequent aggregation studies. To prepare the erythrocytes, the whole blood was centrifuged at 2000×g for 10 min. The plasma and buffy coat were carefully removed to obtain the packed erythrocytes. The erythrocytes were added into PRP to produce different hematocrits. The hematocrit values in the mixture of PRP+erythrocytes were checked by the microhematocrit centrifuge. The PRP+erythrocyte samples were pre-incubated at 37°C with 0.01–10 µM sodium nitrite or DEANONOate for 5 min in the presence or absence of C-PTIO.

The ATP released from platelets was monitored by luminescence aggregometer using a luciferin-luciferase assay. The amount of ATP was calculated from the luminescence signal of ATP standard according to the manufacturing protocol.

### Flow cytometry

The influence of deoxygenation on platelet inhibition by nitrite+erythrocytes was studied by flow cytometry. The erythrocytes were washed three times with an equal volume of PBS and then diluted with PBS to obtain 50% hematocrit. The oxygen saturation in erythrocytes was decreased by helium blown above the cell suspension with gentle stirring for 30 min. As measured by a blood gas analyzer (Stat Profile Critical Care Xpress, Nova Biomedical, Waltham, MA), the deoxygenated erythrocytes contained PO_2_ 25 mmHg and SO_2_ 44% while the erythrocytes at room air contained PO_2_ 57 mmHg and SO_2_ 84%. The deoxygenated erythrocytes were stored in vacuum container at 4°C until used within 12 h.

All reagent solutions were deoxygenated by helium gas. The PRP was diluted 1∶4 (vol/vol) by deoxygenated PBS. 100 µL of the diluted PRP was gently pipetted into a sealed cuvette inside a deoxygenated glovebox. The erythrocytes were added into the cuvette to obtain 20% hematocrit. Afterwards, 0.1 or 1 µM nitrite in the presence or absence of 200 µM C-PTIO was added into the cell suspension. The mixture was incubated at 37°C for 5 min. Then, 20 µM ADP was added into each cuvette and incubated in the mixture for 10 min at 37°C. To prevent further progress of platelet activation, 100 µL of samples were fixed with 1% paraformaldehyde at 4°C for 2 h. 10 µL of sample was added into a tube containing saturated concentration of FITC-labeled CD41a and PE-labeled CD62P. The samples were incubated in dark at room temperature for 15 min and analyzed on a FACScan flow cytometry (Becton Dickinson, Oxford, U.K.). The platelets were distinguished by the characteristic of light scatter and antibody to platelet CD41a. The baseline levels of P-selectin (CD62P) expression, a platelet degranulation marker, were quantified in samples without ADP and used as a reference for subsequent experiments. A percentage of cells with CD62P expression greater than the baseline levels was calculated from 2000 events positive for CD41a.

### Measurement of cGMP levels in platelets

Washed platelets were prepared from PRP [33]. After washing, the pellet was resuspended in the incubation buffer (134 mM NaCl, 12 mM NaHCO_3_, 0.34 mM NaH_2_PO_4_, 2.9 mM KCl, 1 mM CaCl_2_, 0.8 mM MgCl_2_, 5 mM Hepes, 5 mM glucose, pH 7.4). The number of washed platelets was adjusted to 3×10^8^ cells/mL and incubated for 1 h at room temperature before use. 0.1 µM nitrite was added into the platelet suspension in the presence or absence of erythrocytes (20% hematocrit). After incubation with agonist and stirring at 37°C for 5 min, the samples were mixed with 5% trichloroacetic acid and centrifuged at 1500×g for 10 min. The supernatant was collected and stored at −80°C until measurement. The cGMP levels were measured by the enzyme-linked immunoassay (Cayman Chemical, Ann Arbor, MI).

### Measurement of nitrite in blood

The nitrite levels in whole blood, erythrocytes, plasma, and platelets were measured. The fasting venous blood was collected using 3.8% sodium citrate as an anticoagulant. Because nitrite has half-life of 10 min after blood drawing at room temperature, the whole blood were mixed immediately with the nitrite-stabilizing solution and stored at −80°C [29]. In a separate set of experiments, the whole blood was centrifuged immediately at 2000×g for 1 min at 4°C to obtain the packed erythrocytes and plasma. The erythrocytes were immediately mixed with the nitrite-preserving solution within 2 min after blood drawing. The nitrite levels were measured specifically by tri-iodide based chemiluminescence [29], [34] using the chemiluminescence NO analyzer. To measure the nitrite levels in platelets, the whole blood was centrifuged for 120×g at 4°C for 10 min to obtain PRP. The PRP was collected and re-centrifuged at 1500×g to obtain the platelet pellet. The platelet pellet was resuspended in 200 µL of PBS before injection into the tri-iodide solution. The nitrite levels in platelets were calculated following subtraction of the nitrite levels in PBS from the levels in platelet suspension.

### Statistical analysis

All data are means ± SEM. Data processing and statistical analysis were analyzed by GraphPad Prism® version 4 (GraphPad software Inc., San Diego, CA). Correlation was analyzed by Pearson's method. ANOVA with Tukey's multiple comparison were used to compare with acceptable *P*-value<0.05.
